# Round the Hospitals

**Published:** 1920-05-01

**Authors:** 


### ROUND THE HOSPITALS.

The King held an unusually large investiture at Buckingham Palace on April 22, returning to town from Windsor for the occasion. The members of the nursing services who had the honour of receiving their decorations from the hands of His Majesty were as follows:—Royal Red Cross, 1st Class.—Miss Annie Wilson, Q.A.I.M.N.S.; Mrs. Ethel McEwen, Miss Jean Orr, Miss Jane Trotter, Q.A.I.M.N.S. (R.); Miss Elizabeth Kerr, T.F.N.S. Royal Red Cross, 2nd Class: Miss Gladys Parry, Q.A.I.M.N.S.; Miss Elizabeth Beet, Mrs. Jane Howe, Misses Ellie McFadden, Rosina MacMoreland, Edith Porter, Annie Ridley and Ida Tuxford, Q.A.I.M.N.S. (R.); Misses Beatrice Blakeley, Katie Cooper, Winifred Hooper, Battiscombe Mustard and Francis Richardson, T.F.N.S.; Misses

**ROUND THE HOSPITALS—*(continued)*.**
Annie Clapham, Dora Harries and Alice Scruton, C.N.S.; Misses Alice Burfield, Mabel Pepper, Nellie Pickersgill and Leila Poole, B.R.C.S.; Misses Annie Dawson and Ruth Thompson, Civil and War Hospital; Mrs. Nancy Hick and Miss Dorothea Sutherland of Forse V.A.D. All these ladies, together with Miss A. B. Smith, R.R.C., were received at Marlborough House after the Investiture by Queen Alexandra.

---

The event of the week is the announcement by the Ministry of Health of the names of the persons selected to form the First General Nursing Council under the Nurses' Registration Act of 1919. The complete list will be found on page 127. Nurses have on the whole good reason to be satisfied with the choice made by Dr. Addison of members of their own profession to represent them. Out of the fifteen women nurses on the list nine are members of the College of Nursing, while Miss Coulton, Miss Cox-Davies, Miss Lloyd Still, Miss Sparshott and Miss Worsley are closely associated with its management. To them already much progress in nursing affairs is due and their appointment is highly popular. Miss Dowbiggin, Miss Peterkin, Miss B. Smith, Miss Swiss, Miss Villiers and Miss Yapp are acknowledged authorities in their own departments, all very keen workers in their several branches of the profession and abreast with modern developments of nursing. We have before expressed our opinion that present-day experience in the business of carrying on nurse-training schools or organising district and private nursing is the main consideration, and Dr. Addison has shown how largely this principle has guided his choice. All but two or three of his sixteen nurses are now practising their profession.

---

We foresee a possible danger from the very circumstance which lends the General Nursing Council its dignity and influence. Can members of the profession who hold onerous posts in various parts of England be depended on for the necessary regular attendances which can alone ensure successful and peaceful development? We confess we should have seen with satisfaction the appointment of some recently retired matron who could have devoted her entire time to the affairs of the new Council. Doubtless the authorities of the various institutions which have been honoured by the choice of their matron for this responsible work will make special arrangements to facilitate their attendance at meetings. We all know that it is the busy who have most time to devote to business. But members of the Council must be on their guard lest in their single-hearted devotion to the matters in hand they find their feet entangled in toils spread by members less incessantly engrossed in urgent duties than themselves. We would not seem to anticipate difficulties. The nine members of the Council who are to help nurses in regulating their profession have been wisely chosen. And we have every confidence that the course of their deliberations will show that all, without exception, have the welfare of the profession and the good of the public at heart.

---

Miss Sheriff-MacGregor has been appointed Organising Secretary of the College of Nursing, in succession to Miss Cowlin, who has now taken up her duties at Geneva as assistant director of the nursing division of the League of Red Cross Societies. Miss Sheriff-MacGregor was trained at Westminster Hospital, and has had wide experience in her profession, having successively held the posts of Assistant Matron of the Warneford Hospital, Leamington; Home Sister at the Victoria Hospital, Chelsea; and Matron of Paddington Green Children's Hospital. During the war she served under the Joint War Committee in Rumania, Russia, and Siberia. The College of Nursing and its members are to be congratulated on having secured the services of so able a woman in the responsible office of Organising Secretary.

---

The Army cannot yet do without its faithful band of General Service V.A.D. members and officials. These useful people are to be asked to undertake a fresh engagement when their contract runs out on April 30 to serve till October 31 or until their services are no longer required, whichever is the shorter period. In lieu of the Army of Occupation bonus, the weekly rates of pay under the new contracts will be increased as follows: Commandant, 6s. 3d.; superintendents, 5s. to 5s. 6d.; quartermaster, 5s.; members, 2s.

---

The need for a great development of nursing organisation in Central Europe has long been painfully evident, but the number of women in the war-devastated countries fitted to undertake pioneer duties and found training-centres is very limited. The League of Red Cross Societies at Geneva has reached the conclusion that the imperative call at the present moment is to train nurses to undertake this very special class of work. In furtherance of this scheme they offer ten public health nursing scholarships of 1,000 dollars each and travelling expenses, to be available for the Red Cross Societies of the war-stricken countries or of those which wish to improve their organisation. The courses begin in October at King's College for Women, London University, and each graduate must undertake on the completion of her course to devote herself to the initiation and development of public-health nursing in her respective country. The College of Nursing, by whose efforts the nursing courses at King's College were first started, must be gratified at this new international development.

---

The new rates of pay for temporary Army nurses (Q.A.I.M.N.S. Reserve, T.F.N.S., V.A.D. nursing members, and special military probationers) were issued on April 24. They do away with the Army of Occupation bonus and substitute a far more satisfactory scale. Nurses are expected to sign a contract to serve for either one or two years, if so long required, and are to receive no gratuity

ROUND THE HOSPITALS—(*continued*).

on the termination of service. The rates are as follow: Matrons, £120 10s., rising by yearly increment of £10 to £195 10s.; assistant matrons, £120 10s.; sisters, £77 10s., by yearly increment of £5 to £92 10s.; staff nurses, £63, by yearly increment of £2 10s. to £68; V.A.D. nursing members and special military probationers, £30, by half-yearly increment of £2 10s. to £40. Nurses already drawing the additional pay of £20 a year granted in 1916 to such as undertook to serve for so long as required will continue to do so. Allowances, when admissible, will continue to be drawn at the rates laid down for the corresponding ranks of Q.A.I.M.N.S.

It is not generally known what a very great scarcity of trained hospital nurses, available for private work, exists at the present time, and it would be well if the very large number of nurses there are in the country realised more what excellent scope there is in this branch of their work. Many doctors have found it an almost impossible task in London during the last few months to obtain a nurse anywhere immediately, even though she be urgently needed, and it is no uncommon thing to have to telephone to a dozen or more nurses' co-operations and societies before a nurse can be definitely promised. Similar conditions are experienced in other districts—and the distressing reply to the query for a nurse—"We have not a nurse at all," has been a great source of worry both to doctors and the patient's friends. Indeed, the conditions a month or so ago were little if any better than during the acute shortage of the late stages of the war. The scarcity of domestic help in a large measure accounts for a considerably increased demand for nurses, for often in the past the mothers and other members of families would nurse a more simple case themselves, whereas now their time is fully occupied with household duties, and they are forced to obtain the services of a trained nurse when illness comes. There is no doubt, therefore, that private nursing offers greatly improved prospects at the present moment, and the income which may be earned by a well-trained nurse compares more than favourably with many other spheres of women's work. Nursing fees have been raised to three guineas and upwards per week according to the type of case, and this sum, together with full board and expenses, affords possibilities for a nurse to save a very fair amount each year, for under existing conditions a nurse should very rarely be without a "case."

A stirring letter has been sent out by the Chairman of the London County Council "to all fellow-workers in the cause of London education" in support of the appeal for the London Schools Hospital Fund. The appeal has been circulated to every school, private and public, in Greater London, and asks every teacher to make a collection among his pupils on behalf of London hospitals in the way which best suits individual circumstances. The fund will be open from Easter to Whitsuntide, and its promoters hope for a million donors with a total of not less than £50,000. It is an admirable project, and if rightly undertaken may lead to an indefinite expansion of interest in hospital work. What the children are keen about their parents can hardly fail to be interested in, and to set stirring a current of sympathy with hospital work in the mind of the child will surely bear rich fruit in the generation to come.

The Nurses' Home Fund of the Great Northern Hospital is being pressed forward with all enthusiasm. The Marquis of Northampton, Chairman of the hospital, is among those who are working most zealously, and he has enlisted the sympathies of Queen Alexandra on its behalf. The next great effort is a Matinée, which has been arranged to take place at the Palladium on May 14. Queen Alexandra has graciously expressed her hope of being present, and the following artistes have already consented to give their services:—Miss Margaret Cooper, Miss Gladys Cooper, Mr. Gervase Elwes, Mr. Cecil Sherwood, Lady Tree, Miss Irene Vanbrugh.

The proposed Elsie Inglis Memorial is expanding to an extent hardly guessed at in its first inception. It has a double aim, first the extension and endowment of the Maternity and Child Welfare Centre, which Dr. Inglis inaugurated in Edinburgh; and secondly, the foundation of a nurse training-school or college for Serbian women in Serbia. It is now announced that each enterprise will cost at least £50,000. A highly successful effort took place on Shakespeare's birthday, April 23, in aid of the fund, when Mr. Arthur Bourchier lent the Strand Theatre and the "all-woman" caste of *Hamlet*, arranged by the British Empire Shakespeare Society.

It is stated by Mr. Ben Purse, President of the National League of the Blind, that 40 per cent. of the blind have lost their sight after they had turned thirty-five years of age, many by reason of the dangerous character of their employment. The percentage blinded within six months of their birth is 21, and it is clear that a comprehensive inquiry on scientific lines into the causes and prevention of injuries to sight, such as Dr. Addison has decided on, would lead to a great decrease in the numbers of the blind in the next generation. However desirable it may be to provide institutions for certain disabled persons, for the blind such confinement, which too often means hopeless inaction, is nearly always a calamity, and we trust this fact will be kept well before the public. If by the aid of a pension the blind can be stimulated to use their brains and hands the comparatively small subsidy required will be grudged by none.

The organisers of nearly all the beds at the Elizabeth Garrett Anderson Hospital, for which endow-

**ROUND THE HOSPITALS**—(*continued*).

ment was asked in memory of the great foundress, have now made up the required sum of £1,000. The latest to be completed is the Stage Bed, which was undertaken by Miss Irene Vanbrugh as organiser. Miss Vanbrugh's entertainment on April 20 at Dudley House, kindly lent by Lady Dudley for the purpose, was brilliantly successful, and put the finishing touch to the fund. The policy of enlisting the services of various groups of persons to make themselves responsible for a bed has proved very stimulating to givers, and indicates a path which might be followed elsewhere to advantage.

---

The Linen League of St. Bartholomew's Hospital, Rochester, has proved once again that these useful auxiliaries can often lead the way in securing support for the institutions to which they are attached. At Lower Halslow, one of the villages affiliated, inhabited wholly by working people, a fête was held under the auspices of the League, and no less than £163 was cleared. A goodly stock of sheeting and blankets was purchased, together with material for undergarments sufficient to keep the workers busy throughout the year for the hospital. The men in the village are as keen as the women, and a large collecting-box bearing the words "The ladies are giving their spare time for working, you give your spare cash" proves an excellent stimulus. All this shows that wherever the right methods are adopted people, rich and poor alike, take a pride in their hospital and give to it with real pleasure.

---

Whatever successes the Bolshevists may boast of in their Soviet administration, it is certain they have not so far managed to give even the most elementary care to the sick or set up any efficient sanitary organisation. Both in Moscow and Petrograd typhus is raging, and the disease is slaying hundreds of thousands all over the country. In the whole region of the Don vermin in human habitations have become an unbearable pest. The towns and villages stink with putrefying filth and offal. Diphtheria and spinal and cerebral meningitis are making fearful ravages among the children. Vast hospitals have been commenced, but cannot be equipped, and there are neither doctors nor nurses. All honour to Lady Muriel Paget and her band of devoted workers, who are devoting themselves to provide a chain of hospital units through the most distressed parts of Russia. In Dwinsk alone, she reports, there are 40,000 destitute people and about 10,000 children. Mr. Webster is there in charge of the mission with a vast work confronting him. Lady Muriel Paget is working in close touch with the League of Red Cross Societies and the "Save the Children" Fund.

---

Streatham is leading the way in the matter of converting war activities to the uses of peace. Its fine little Red Cross hospital in the Christ Church Road, which was opened in 1916 and has been doing excellent work ever since, has now been bought by the Streatham War Hospital Supply Depôt and secured for the use of residents as a medical and surgical nursing home. It was opened on April 17 by the County Director Major-General Sir Richard Ewart, who was accompanied by the Mayor and Mayoress of Wandsworth, the Rector of Streatham, and numerous other officials. It is proposed to staff the home hospital largely by means of voluntary workers, members of the detachments which proved their worth during the war. It is intended for patients unable to pay ordinary nursing-home fees, and £3 3s. a week will be the maximum charge, while in cases of necessity this fee will be greatly reduced. The experiment will be watched with considerable interest. It is, we believe, the first of its kind, and if successful should pave the way for similar undertakings, and ought to have the effect of relieving the present congested state of the hospitals and lessening the long waiting-lists.

---

The North London Nursing Association gives a pleasantly grouped portrait of its eleven nurses as frontispiece to the annual report. During the war the staff declined to four nurses, and the Committee express their warm gratitude to the Superintendent, Miss Wills, through whose exertions it was found possible to carry on, and their appreciation of her devoted work during the past year. The work of the nurses has been considerably enlarged since the care of measles patients has been added, but it is highly satisfactory to learn that the mortality from measles has been greatly reduced since this arrangement was made. The excess of expenditure over income for the year was £133.

---

At Southend Corporation Health Committee the medical officer of health reported that he had had to suspend a midwife from practice for ten days in order to prevent the spread of the disease known as pemphigus neonatorum, and that upon resuming duty it had been necessary to suspend her again. As her suspension was not brought about by her own neglect the Health Committee proposes to give her compensation at the rate of £3 a week.

---

Immense sympathy all along the lines of route has been roused by the march of the civilian blind since Easter from far and wide to London, which they reached on April 25. Their movements were cleverly planned in advance, so that the men from the West and from Wales met at Birmingham and from the Eastern Counties at Leicester, while contingents from the North, gathering at Leeds and Manchester, they eventually became one body, numbering in all some 250. They have come to plead with the Prime Minister the cause of the sightless, of whom there are 35,000, men and women, in the United Kingdom. In London alone there are 3,552 blind persons, a third of whom are confined in institutions.

